# Disparities in Survival Due to Social Determinants of Health and Access to Treatment in US Patients With Operable Malignant Pleural Mesothelioma

**DOI:** 10.1001/jamanetworkopen.2023.4261

**Published:** 2023-03-23

**Authors:** Ahmed Alnajar, Samuel A. Kareff, Syed S. Razi, J. Sunil Rao, Gilberto De Lima Lopes, Dao M. Nguyen, Nestor Villamizar, Estelamari Rodriguez

**Affiliations:** 1 Division of Cardiothoracic Surgery, Department of Surgery, University of Miami Leonard M. Miller School of Medicine, Miami, Florida; 2 Jackson Memorial Hospital, University of Miami Leonard M. Miller School of Medicine, Miami, Florida; 3 Sylvester Comprehensive Cancer Center, University of Miami Leonard M. Miller School of Medicine, Miami, Florida; 4 Division of Thoracic Surgery, Memorial Healthcare System, Hollywood, Florida; 5 Division of Biostatistics, Department of Public Health Sciences, University of Miami Leonard M. Miller School of Medicine, Miami, Florida; 6 Department of Medical Oncology, Sylvester Comprehensive Cancer Center, University of Miami, Miami, Florida; 7 Division of Medical Oncology, Department of Internal Medicine, University of Miami Leonard M. Miller School of Medicine, Miami, Florida

## Abstract

**Importance:**

Outcomes of localized malignant pleural mesothelioma (MPM) remain poor despite multimodality therapy. It is unclear what role disparities have in the overall survival (OS) of patients with operable MPM.

**Objective:**

To examine survival disparities associated with social determinants of health (SDOHs) and treatment access in patients with malignant pleural mesothelioma.

**Design, Setting, and Participants:**

In this observational, retrospective cohort study, patients with MPM diagnosed between January 1, 2004, and December 31, 2017, were identified from the National Cancer Database with a maximum follow-up time of 13.6 years. The analysis was conducted from February 16, 2022, to July 29, 2022. Patients were included if they were diagnosed with potentially resectable clinical stage I to IIIA MPM, had epithelioid and biphasic histologic subtypes, and received chemotherapy. Patients were excluded if they could not receive curative surgery, were 75 years or older, or had metastasis, unknown stage, or tumor extension to the chest wall, mediastinal tissues, or organs.

**Exposures:**

Chemotherapy alone vs chemotherapy with curative surgery in the form of pleurectomy and decortication or extrapleural pneumonectomy.

**Main Outcomes and Measures:**

The primary end point was OS. Cox proportional hazards regression models were used to determine hazard ratios (HRs) for OS, including univariable and multivariable models controlling for potential confounders, including demographic, comorbidity, clinical, treatment, tumor, and hospital-related variables, as well as SDOHs.

**Results:**

A total of 1389 patients with MPM were identified (median [IQR] age, 66 [61-70] years; 1024 [74%] male; 12 [1%] Asian, 49 [3%] Black, 74 [5%] Hispanic, 1233 [89%] White, and 21 [2%] of other race). The median OS was 1.7 years (95% CI, 1.6-1.8). Risk factors associated with worse OS included older age, male sex, Black race, low income, and low educational attainment. Factors associated with greater odds of survival included receipt of surgical therapy, recent year of treatment, increased distance to travel, and treatment at high-volume academic hospitals. The risk factors most strongly associated with poor OS included Black race (HR, 1.96; 95% CI, 1.43-2.69) and male sex (HR, 1.60; 95% CI, 1.38-1.86). Surgical treatment in addition to systemic chemotherapy (HR, 0.70; 95% CI, 0.61-0.81) was independently associated with improved OS, as were chemotherapy initiation (HR, 0.93; 95% CI, 0.87-0.99) and greater travel distance from the hospital (HR, 0.92; 95% CI, 0.86-0.98).

**Conclusions and Relevance:**

In this retrospective cohort study of patients with operable MPM, there was significant variability in access to care by SDOHs. Addressing disparities in access to multimodality therapy can help ensure equity of care for patients with MPM.

## Key Points

**Question:**

What is the association of social determinants of health (SDOHs) with treatment and survival of patients with malignant pleural mesothelioma?

**Findings:**

In this nationwide cohort study of 1389 adults with operable mesothelioma, surgery was associated with improvement in overall survival. However, SDOHs significantly attenuated these findings, with the greatest risk factors being older age, male sex, Black race, low income, and low educational attainment.

**Meaning:**

These findings suggest that although surgical therapy in mesothelioma may prolong survival, SDOHs may have a negative association with survival.

## Teaser

This cohort study examines patterns of care and overall survival among patients with operable malignant pleural mesothelioma by treatment access and social determinants of health.

## Introduction

Malignant pleural mesothelioma (MPM) represents a relatively rare histologic tumor type both in the US and globally. It accounts for less than 0.17% (approximately 3000 total) of all US tumor diagnoses as of 2018, and this proportion is expected to continue decreasing given greater awareness of causality linked to asbestos exposure.^1^ Guidelines suggest that multimodality therapy, including surgery, chemotherapy, and possible radiation therapy, can lead to the best possible outcomes for this rare tumor type.^2^ Unfortunately, outcomes of localized MPM remain poor despite multimodality therapy.

Nonclinical factors may play a role in these findings; however, data are limited regarding the impact of disparities in access to care and patients’ social determinants of health (SDOHs) in disease-related outcomes for patients with potentially operable MPM. A recent review^3^ demonstrated that lower income and lack of insurance are associated with worse survival within the US and globally; however, there is no unifying definition of income level on which to define this observation. Similar findings related to median zip code income have been reported for surgical treatment in early-stage lung cancer.^4^ Moreover, additional SDOHs, such as level of education, have yet to be evaluated systematically in MPM, although they have been identified in other thoracic tumor types, such as tracheal cancer.^5^ Furthermore, although the distance to treatment is associated with survival rates in multiple reports, access to treatment for patients with MPM should be studied. Our objective was to examine factors associated with patterns of care as well as overall survival (OS) among patients with operable MPM by treatment access and patients’ SDOHs.

## Methods

### Data Source

The data used in this cohort study are derived from a deidentified 2017 National Cancer Database (NCDB) participant user data file. The NCDB is a hospital-based cancer registry that is a joint program of the American College of Surgeons Commission on Cancer and the American Cancer Society. The NCDB annually collects data from more than 1500 Commission on Cancer–accredited hospitals in the US, capturing approximately 70% of cancer diagnoses annually. The NCDB data are collected by electronic medical record review by trained abstractors. Race and/or ethnicity is recorded in the patient’s medical record; however, each participating institution may document race and/or ethnicity in the medical record by different means, which are not recorded by the NCDB. The University of Miami Leonard M. Miller School of Medicine Institutional Review Board deemed this study exempt from review and the need for informed consent because the data were deidentified. This study followed the Strengthening the Reporting of Observational Studies in Epidemiology (STROBE) reporting guideline.

### Study Population

This study included adult (age >18 years) patients diagnosed with MPM between January 1, 2004, and December 31, 2017, with a follow-up time of up to 13.6 years. The analysis was conducted from February 16, 2022, to July 29, 2022. Inclusion criteria included diagnosis with clinical stage I to IIIA (ie, potentially resectable) MPM, epithelioid and biphasic mesothelioma histologic subtypes, and receipt of chemotherapy with or without curative surgery. Surgical treatment was defined as pleurectomy and decortication defined by NCDB codes 20:23, 30, 33, 40, 45:48, and 50 and extrapleural pneumonectomy defined by codes 55:56, 60, 66, and 70. Exclusion criteria included any patient who received palliative surgery and those who did not receive surgery due to contraindication related to patient risk factors or oncologist recommendation; age of 75 years or older; and patients with reported metastasis, unknown stage, unreported follow-up status, or tumor extension that involved the chest wall, mediastinal tissues, or organs (eTable 1 in Supplement 1). The study included patients with MPM who underwent exposure to either surgical treatment with chemotherapy or chemotherapy only. The survival outcomes were obtained from the follow-up data reported in the NCDB registry.

### Study Outcomes and Covariates

The primary outcome of interest was OS, which was measured as the time from diagnosis to the date of last contact or death (from any cause). The SDOHs included zip code–level median income (<100% federal poverty level [FPL], 100%-150% FPL, or >150% FPL based on the neighborhood of patient residence derived from 2016 US Census data), metropolitan statistical area (metropolitan, urban, or rural), educational attainment (based on a percentage of individuals holding high school degrees according to zip code), and distance between the patient’s residence and the hospital that reported the case. Travel distance to cancer treatment was defined as the driving distance between the geographic centroid of zip codes of patient residence at diagnosis and the reporting facility, which was categorized based on previous analyses as 0 to 12.49, 12.5 to 249, and 250 or more miles.^6^ Other variables of interest included demographic variables, such as age, sex (male or female), race and/or ethnicity (grouped into Hispanic, non-Hispanic Black, non-Hispanic White, or other [other than Hispanic, non-Hispanic Black, or non-Hispanic White]), Charlson-Deyo Comorbidity Index (0 or ≥1), facility type (academic, integrated, and community), and facility case volume (disease-specific, categorized into high and low by the median yearly case volume [median, 4 annual cases]).

### Statistical Analysis

Descriptive statistics were used to summarize patient characteristics. Wilcoxon rank-sum, Pearson χ^2^, and Fisher exact tests were used to determine the significance of differences in patients who received chemotherapy with and without curative surgical treatment. For the descriptive analyses, the missing values of each variable were presented in a separate category in their corresponding tables. The Kaplan-Meier method estimated OS, which was compared between different variables by using the log-rank test. A Cox proportional hazards regression was used to evaluate the association between patient characteristics and access to care (ie, SDOHs and facility-related variables) on OS in univariable models. A multivariable Cox proportional hazards regression model was used to account for patient-, tumor-, treatment-, and hospital-level variables after data imputation (by mode for categorical variables and mean for continuous variables), variable correlation (by variance inflation factor), proportional hazards assumption assessment (by Schoenfeld residuals), and *z* score transformation for all continuous variables. Available covariates were then incorporated into the model. Two-sided *P* values were reported along with 95% CIs and were considered significant at *P* < .05. All statistical analyses were performed with R software, version 4.2.2 with survminer and gtsummary packages, along with their dependencies (R Foundation for Statistical Computing).

## Results

### Study Cohort Overview

#### Patient Characteristics

A total of 1389 patients (median [IQR] age, 66 [61-70] years; 1024 [74%] male) were identified from the NCDB as being diagnosed with MPM from January 1, 2004, to December 31, 2017. Most patients were non-Hispanic White (1233 [89%]), whereas 12 (1%) were Asian, 49 (4%) were Black, 74 (5%) were Hispanic, and 21 (2%) were of other race. There were no significant racial or ethnic differences between patients who did or did not receive surgery; however, Race disparities were evident because Black patients were 80% more likely to die than White patients. A total of 727 patients (52%) had Medicare or other governmental insurance, whereas 571 (42%) held private insurance (Table 1). Among SDOHs, only 578 patients (44%) were living in areas with incomes greater than 150% FPL, 1107 (78%) lived in metropolitan areas, and 910 (66%) lived in areas with low educational attainment. We identified an 18% increased likelihood of survival in patients living in areas with a median income greater than $63 333 (>150% FPL) compared with those living in lower-income areas (≤150% FPL).

**Table 1.** Characteristics of the Study Patients and Hospitals Indexed in the National Cancer Database (2004-2017) by Treatment Modality^a^

Characteristic	Overall (N = 1389)	Chemotherapy only (n = 674)	Chemotherapy and surgery (n = 715)	*P* value^b^
Age, median (IQR), y	66 (61-70)	67 (63-71)	65 (59-70)	<.001
Age category, y				
18-45	37 (3)	11 (2)	26 (4)	<.001
45-54	108 (8)	39 (6)	69 (10)	
55-64	434 (31)	177 (26)	257 (36)	
≥65	810 (58)	447 (66)	363 (51)	
Sex				
Female	365 (26)	167 (25)	198 (28)	.22
Male	1024 (74)	507 (75)	517 (72)	
Race and ethnicity^c^				
Hispanic	74 (5)	36 (5)	38 (5)	.35
Non-Hispanic				
Asian	12 (1)	4 (1)	8 (1)	
Black	49 (3)	29 (4)	20 (3)	
White	1233 (89)	600 (89)	633 (89)	
Other	21 (2)	5 (1)	16 (2)	
Income				
>150% FPL	578 (42)	235 (36)	343 (52)	<.001
100%-150% FPL	556 (42)	304 (47)	252 (38)	
<100% FPL	177 (14)	111 (17)	66 (10)	
Unknown	78	24	54	
Geographic setting				
Metropolitan	1107 (85)	531 (82)	576 (88)	.007
Urban	169 (13)	103 (16)	66 (10)	
Rural	29 (2)	14 (2)	15 (2)	
Unknown	84	26	58	
Educational attainment				
High	479 (34)	204 (30)	275 (38)	.001
Low	910 (66)	470 (70)	440 (62)	
Insurance status				
Private insurance	571 (41)	211 (32)	360 (50)	<.001
Medicaid or not insured	52 (4)	32 (5)	20 (3)	
Medicare or other government	727 (52)	409 (63)	318 (45)	
Unknown	39	22	17	
Years of diagnosis				
2004-2008	289 (21)	185 (27)	104 (15)	<.001
2009-2013	650 (47)	307 (46)	343 (48)	
2014-2017	450 (32)	182 (27)	268 (37)	
Charlson-Deyo Comorbidity Index				
0	999 (72)	464 (69)	535 (75)	.01
1	293 (21)	149 (22)	144 (20)	
2	76 (6)	46 (7)	30 (4)	
3	21 (2)	15 (2)	6 (1)	
Hospital volume				
High	822 (59)	314 (47)	508 (71)	<.001
Low	567 (41)	360 (53)	207 (29)	
Facility type				
Academic	760 (55)	266 (39)	494 (69)	<.001
Community	477 (35)	324 (48)	153 (22)	
Integrated	134 (10)	80 (12)	54 (8)	
Unknown	18	4	14	
Facility distance, median (IQR), miles^d^				
Total	18 (7-47)	12 (5-33)	21 (9-72)	<.001
Unknown	76	22	54	
Great circle distance, miles				
0-12.49	557 (42)	327 (50)	230 (35)	<.001
12.5-249	682 (52)	310 (48)	372 (56)	
≥250	74 (6)	15 (2)	59 (9)	
Unknown	76	22	54	

Abbreviation: FPL, federal poverty level.

^a^ This table gives patients’ individual and social determinants of health characteristics indexed in the National Cancer Database from 2004 through 2017 and stratified by surgery as a part of treatment plan (overall, chemotherapy only, and chemotherapy with surgery). Data are presented as number (percentage) of patients unless otherwise indicated. Missing (unknown) data were not included in calculating the percentages. Some percentages may not equal 100.

^b^ Wilcoxon rank-sum test, Pearson χ^2^ test, or Fisher exact test.

^c^ Hispanic ethnicity was measured in addition to Asian, Black, White, or other races according to the National Cancer Database; totals may be greater than 100%. Other indicates race and ethnicity other than Hispanic, non-Hispanic Black, or non-Hispanic White.

^d^ Facility distance from the center of the patients’ zip code was based on the great circle distance (miles).

#### Tumor Characteristics

A total of 620 patients (45%) had clinical stage I MPM, whereas 423 patients (31%) had stage II and 337 (24%) had stage III MPM. Patients with clinical stage I disease were less likely to receive surgery than chemotherapy alone (348 [56%] vs 272 [44%]), whereas patients with stage II disease (187 [44%] vs 236 [56%]) and stage III disease (135 [40%] vs 202 [60%]) were more likely to receive surgery. However, all patients with pathologic stage disease were more likely to undergo surgery (eTable 2 in Supplement 1). Once pathologic staging was performed, stage IV MPM was discovered in 49 patients (8%), mostly in those who received curative surgery. Epithelioid mesothelioma was the most common histologic subtype (1141 [82%]), followed by biphasic mesothelioma (248 [18%]) (eTable 3 in Supplement 1).

#### Treatment and Facility Characteristics

Overall, most operations were provided at high-volume (822 [59%]) and academic (760 [55%]) facilities. Compared with patients who did not receive surgery, patients who received surgery traveled a greater distance for treatment (median [IQR], 21 [9-72] miles; *P* < .001) and received treatment at an academic (494 [69%] vs 266 [39%], *P* < .001) or higher-volume facility (508 [71%] vs 314 [47%], *P* < .001) than those who received chemotherapy alone. Patients who received surgery had delayed chemotherapy initiation for more days after their diagnosis (median [IQR], 57 [36-92] vs 40 [27-64] days; *P* < .001). Curative operations were mostly performed through an open approach rather than a minimally invasive surgical approach (mainly video-assisted thoracoscopic surgery). Other additional innovative and experimental treatments, such as immunotherapy, were not different between the groups (eTable 4 in Supplement 1).

### Overall Survival

**Figure 1.**
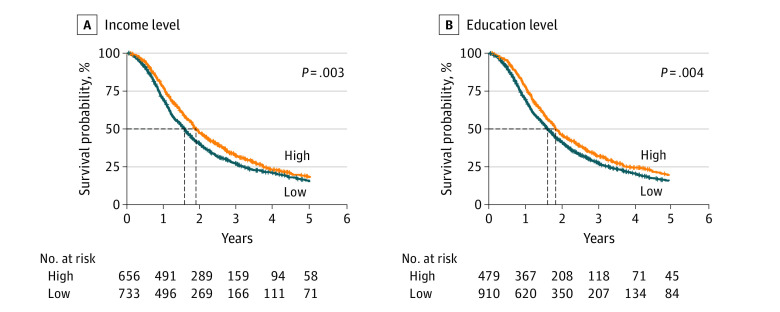
Comparison of Income and Education Levels of the Study Patients

This cohort’s 6-month OS was 92% (95% CI, 91%-94%), which was higher in patients who underwent curative surgery (96%; 95% CI, 94%-97%) vs those who did not (88%; 95% CI, 86%-91%). Overall survival was 73% at 1 year (81% vs 64%), 29% at 3 years (38% vs 20%), and 17% at 5 years (24% vs 9.1%). The median OS was 1.7 years (95% CI, 1.6-1.8): 2.2 years (95% CI, 2.0-2.4 years) for patients who received curative surgery compared with 1.3 years (95% CI, 1.2-1.5 years) for those who did not (eFigure 1A in Supplement 1). The median survival time for operations performed at centers more than 250 miles away was 3.9 years (95% CI, 2.6 years to not estimable) compared with 2.1 years (95% CI, 1.9-2.3 years) for centers less than 250 miles away (Figure 1).

Factors associated with OS are summarized as follows. The risk factors most strongly associated with poor OS included Black race (hazard ratio [HR], 1.96; 95% CI, 1.43-2.69) and male sex (HR, 1.60; 95% CI, 1.38-1.86). Moreover, Hispanic patients in our study were 13% less likely to die, but this finding was not significant when compared with the mortality rate of White patients. In unadjusted Cox proportional hazards regression analyses, there appeared to be an association between decreased income (HR, 1.19; 95% CI, 1.06-1.34; *P* = .003) and educational attainment (HR, 1.21; 95% CI, 1.06-1.37; *P* = .004) levels with overall poor OS (Figure 1). Increased travel distance was associated with better OS (HR, 0.86; 95% CI, 0.81-0.92; *P* < .001), whereas community (HR, 1.53; 95% CI, 1.34-1.73; *P* < .001) and low-volume (HR, 1.25; 95% CI, 1.11-1.41; *P* < .001) facilities demonstrated decreased odds of OS (eTable 5 in Supplement 1).

After adjustment for relevant patient and tumor characteristics, as well as SDOHs and facility-related variables, income (HR, 1.01; 95% CI, 0.87-1.17; *P* = .90) and educational attainment (HR, 1.12; 95% CI, 0.96-1.31, *P* = .15) were no longer statistically significant. However, decreased travel distance (HR, 0.92; 95% CI, 0.86-0.98; *P* = .006) and receipt of treatment at nonacademic facilities (HR, 1.18; 95% CI, 1.01-1.37; *P* = .03) remained independently associated with worse OS. In addition, hospital volume was not a significant factor (HR, 0.94; 95% CI, 0.81-1.09; *P* = .39). Clinical stage III became a statistically significant risk factor (HR, 1.21; 95% CI, 1.04-1.42; *P* = .02). Curative surgical treatment was associated with a 30% increased likelihood of survival as a part of multimodal therapy (HR, 0.70 CI: 0.61-0.80, *P* < .001), and chemotherapy initiation was associated with a 7% improvement in OS (HR, 0.93; 95% CI, 0.87-0.99). Older age, female sex, median days from diagnosis to treatment with chemotherapy, and more recent year of diagnosis were independently associated with improved OS (Table 2).

**Table 2.** Multivariable Cox Proportional Hazards Regression (Adjusted) Model for Factors Associated With Overall Survival^a^

Characteristic	HR (95% CI)	*P* value
Age at diagnosis (*z* score)^b^	1.13 (1.04-1.23)	.003
Year of diagnosis (*z* score)^b^	0.91 (0.85-0.97)	.002
Sex		
Female	1 [Reference]	
Male	1.60 (1.38-1.86)	<.001
Black race	1.96 (1.43-2.69)	<.001
Income <150% FPL	1.01 (0.87-1.17)	.90
Low educational attainment	1.12 (0.96-1.31)	.15
Insurance status		
Private insurance	1 [Reference]	
Medicaid or not insured	1.31 (0.93-1.84)	.12
Medicare or other government	1.10 (0.94-1.29)	.22
Charlson-Deyo Comorbidity Index >0	0.96 (0.84-1.10)	.59
Clinical stage		
I	1 [Reference]	
II	1.06 (0.92-1.22)	.40
III	1.21 (1.04-1.42)	.02
Therapy modality		
Chemotherapy alone	1 [Reference]	
Chemotherapy and surgery	0.70 (0.61-0.80)	<.001
Chemotherapy initiation	0.93 (0.87-0.99)	.02
Hospital volume		
High	1 [Reference]	
Low	0.94 (0.81-1.09)	.39
Distant facility (*z* score)^b^	0.92 (0.86-0.98)	.006
Nonacademic hospitals	1.18 (1.01-1.37)	.03

Abbreviations: FPL, federal poverty level; HR, hazard ratio.

^a^ This table gives findings of a multivariable Cox proportional hazards regression model analysis related to prognostic factors on overall survival of patients with malignant pleural mesothelioma indexed in the National Cancer Database from 2004 through 2017. After adjustment, the statistically significant factors included age, year of diagnosis, male sex, Black race, distant facility, nonacademic facilities, clinical stage III, receipt of chemotherapy and surgery, and delayed chemotherapy initiation.

^b^ *z* score transformation = (value − mean)/SD.

## Discussion

**Figure 2.**
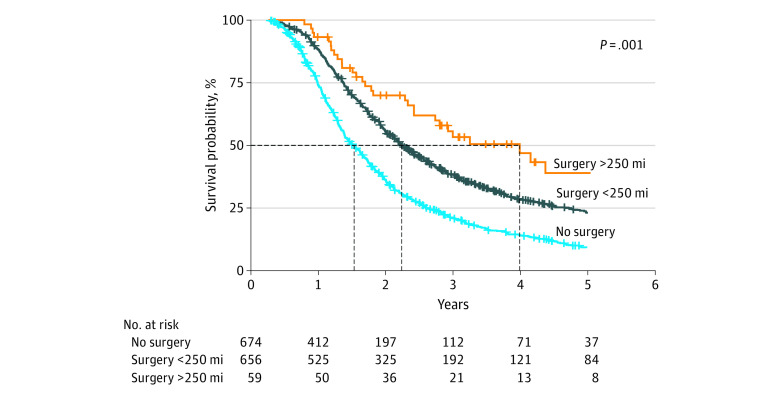
Comparison of Patients Who Underwent Curative Surgery in Distant and Close Facilities to Their Homes vs All Patients With Chemotherapy Without Curative Surgery

The findings of this cohort study suggest that patient population groups with limited access to the most specialized cancer care settings are at increased risk of mortality. Although access to health care services may be associated with individual- or community-level demographic factors, such as race and ethnicity, income level, educational attainment, and health insurance status, geospatial factors affect access to specialized services that are not equitably distributed geographically. Because curative surgery, especially extrapleural pneumonectomy, requires experienced specialized surgical teams in large medical facilities that may not be near the residence of patients with MPM, greater travel distance was positively associated with patients’ survival (Figure 2). Our results echoed previous research dedicated to this topic in other forms of cancer.^7,8^ Although geographic distance imposes an increased travel burden, the benefits may outweigh the costs by enabling more patients to receive optimal treatment, as seen in a previous study^9^ of surgical oncology patients.

Curative surgical treatment reduced the likelihood of mortality by 30%, with a clear separation of survival curves over time (eFigure 1A in Supplement 1). There was no difference in survival between extrapleural pneumonectomy and pleurectomy and decortication in our cohort (eFigure 1B in Supplement 1). These improved outcomes from undergoing surgery, which remained significant even after controlling for patients’ race, SDOHs, and tumor characteristics, highlight the role of multimodality treatment, similar to what was previously recommended for localized disease.^10^ Surgical treatment was additionally found to be predominantly performed at academic and high-volume facilities. Those patients who underwent surgical curative treatment at these facilities also demonstrated delayed chemotherapy initiation, which may serve as a surrogate of adherence to guideline-concordant care, because chemotherapy timing was associated with a 7% improvement in OS.

In addition to the variability in access to care as measured by distance to facility or facility type and/or volume, it was notable that an urban-rural divide may play a role. Our sample has too few patients living in rural areas to draw a meaningful conclusion in patients with MPM, similar to what has been demonstrated in other tumor types.^4^ However, previous suggestions of combining initial diagnostics locally with coordination and continuity of care at referral facilities may mitigate some of the access to care disparities revealed in our analysis.^11^ Policy makers and health care system architects should continue addressing disparities in access to surgery and multimodality therapy to ensure equity of care for patients with MPM.

Our findings also support previous results that highlight the important association of individual characteristics with cancer outcomes and survival. Race disparities were evident, because Black patients were 80% more likely to die than White patients. When compared with other racial and ethnic groups and after adjusting for important covariates, this mortality risk increased remarkably. Previous reports^1,12^ indicate that Black patients are less likely to undergo extensive curative surgery. Although in our cohort patients’ race and ethnic backgrounds were not different between patients who underwent curative surgery vs those who did not (probably because of the robust inclusion and exclusion criteria), our survival results are consistent with findings from previous studies.^12,13^ The mortality rate for Hispanic patients was not different from that of non-Hispanic White patients, and Hispanic patients had a higher survival rate than other racial and ethnic groups, although this difference did not reach statistical significance (eFigure 2 in Supplement 1). This finding could be due to the Hispanic paradox, which indicates that Hispanic individuals can have higher life expectancy than non-Hispanic individuals despite low socioeconomic status and insurance as described in other thoracic cancers.^14,15^ Our study included only 7 patients of Hispanic ethnicity; however, additional studies with larger cohorts may confirm whether a Hispanic paradox is true in the case of MPM and survival.

A recent review^3^ demonstrated that age, male sex, White race, lack of insurance, and lower income level have been associated with worse cancer-related survival within the US and globally. Similarly, our results demonstrated that age and male sex are independently associated with poor OS. A previous study^2^ found that octogenarians are less likely to receive care concordant with national MPM guidelines secondary to poorer performance status. Male sex has also been associated with poorer prognosis in previous population-based analyses.^16^ Additional disparities elucidated in our study sample include more male than female as well as more White vs non-White patients receiving surgery as a part of their multimodality treatment. These findings related to sex and race have been similarly explored previously.^1,17^

Interestingly, we identified an 18% increased likelihood of survival in patients living in areas with a median income greater than $63 333 (>150% FPL) compared with those living in lower-income areas (≤150% FPL). However, this finding was evident only in our unadjusted analysis, which was similar to the 16% (HR, 0.84, *P* < .001) adjustment survival that has been reported in patients with MPM living in areas with similar income levels.^18^ These findings may evolve as treatment paradigms continue maturing to include trimodality therapy for earlier cases.

### Limitations

Although the NCDB includes the broad catchment of MPM cases at the national level, standardized reporting of data among all participating facilities, and rare tumor types, there are certain limitations. Potential limitations include already diagnosed cases included in the NCDB vs a greater number of cases that might have been captured in a more inclusive database, such as the Surveillance, Epidemiology, and End Results database. This limitation could be because NCDB data depend on the participant hospitals, which could introduce a selection bias, because the choice of hospitals depends on different factors. Furthermore, only 198 patients received radiotherapy as part of their treatment plan between patients with (n = 170) and without (n = 28) surgical therapy, limiting our ability to detect a significant association of full multimodal therapy in patients with MPM. In addition, the lack of longitudinal treatment data and clinically relevant end points, such as recurrence and complications, limits our ability to assess patients’ quality of life and disease-free survival. In addition, there could be unmeasured confounding because of the lack of information pertaining to cardiopulmonary status, specific comorbidities, smoking status, the number of surgeons present at a hospital, individual surgeon training, and access to follow-up care.

## Conclusions

In this retrospective cohort study of operable MPM, we found that SDOHs may be associated with suboptimal adherence to national guidelines for the treatment and referral of MPM, particularly in low-volume, nonacademic settings. Adherence to recommended surgery-based multimodal therapy is associated with an overall survival improvement. Key socioeconomic disparities and SDOHs remain to be studied in adjunct to multimodality therapy when treating MPM in the US.

## Notes

Supplement 1. **eTable 1.** The Description and Characteristics of Excluded Patients **eTable 2.** Characteristics of MPM Tumors by Treatment Modality (Row-wise Percentages) **eTable 3.** Characteristics of MPM Tumors by Treatment Modality (Column-wise Percentages) **eTable 4.** Treatment Specifications and Adjunct Treatments Stratified by the Treatment Modality **eTable 5.** Univariable Cox (Unadjusted) Model for Factors Correlated with Overall Survival **eFigure 1.** Kaplan-Meier Survival Curves Comparing Patients Based on Treatment Options and Surgical Types **eFigure 2.** Kaplan-Meier Survival Curves Comparing Patients Based on Race and Ethnicity

Supplement 2.
Data Sharing Statement
