# Supplementary material for: Disparities in Survival Due to Social Determinants of Health and Access to Treatment in US Patients With Operable Malignant Pleural Mesothelioma
Source: JAMA Netw Open. 2023 Mar 23;6(3):e234261. doi: 10.1001/jamanetworkopen.2023.4261 (PMC10037156; doi:10.1001/jamanetworkopen.2023.4261)
Supplement: Supplement 1. — eTable 1. The Description and Characteristics of Excluded Patients eTable 2. Characteristics of MPM Tumors by Treatment Modality (Row-wise Percentages) eTable 3. Characteristics of MPM Tumors by Treatment Modality (Column-wise Percentages) eTable 4. Treatment Specifications and Adjunct Treatments Stratified by the Treatment Modality eTable 5. Univariable Cox (Unadjusted) Model for Factors Correlated with Overall Survival eFigure 1. Kaplan-Meier Survival Curves Comparing Patients Based on Treatment Options and Surgical Types eFigure 2. Kaplan-Meier Survival Curves Comparing Patients Based on Race and Ethnicity [file jamanetwopen-e234261-s001.pdf]

Supplementary Online Content

Alnajar A, Kareff SA, Razi SS, et al. Disparities in survival due to social determinants of health and access to treatment in US patients with operable malignant pleural mesothelioma. *JAMA Netw Open*. 2023;6(3):e234261. doi:10.1001/jamanetworkopen.2023.4261

eTable 1. The Description and Characteristics of Excluded Patients

eTable 2. Characteristics of MPM Tumors by Treatment Modality (Row-wise Percentages)

eTable 3. Characteristics of MPM Tumors by Treatment Modality (Column-wise Percentages)

eTable 4. Treatment Specifications and Adjunct Treatments Stratified by the Treatment Modality

eTable 5. Univariable Cox (Unadjusted) Model for Factors Correlated with Overall Survival

eFigure 1. Kaplan-Meier Survival Curves Comparing Patients Based on Treatment Options and Surgical Types

eFigure 2. Kaplan-Meier Survival Curves Comparing Patients Based on Race and Ethnicity

This supplementary material has been provided by the authors to give readers additional information about their work.

© 2023 Alnajar A et al. *JAMA Network Open.*

eTable 1: The Description and Characteristics of Excluded Patients.

Excluded patients	
Age < 18 or > 75	18,226 (55%)[1]
Non-epithelioid histology	20,371 (62%)
Non-biphasic histology	30,711 (93%)
Unknown Stage	24,325 (73%)
Clinical Stage IV	9,751 (29%)
Metastases	5,332 (16%)
No surgery or chemotherapy	16,758 (51%)
Death prior to surgery	100 (0.3%)
Unknown Follow up	2,464 (7.4%)
Surgery not recommended	1,686 (5.1%)
Tumor extension to mediastinum, rib, or heart tissues	4,548 (14%)

[1]n (%)

© 2023 Alnajar A et al. *JAMA Network Open.*

eTable 2: Characteristics of MPM Tumors by Treatment Modality (Row-wise Percentages).

**Characteristic**	**Chemo Only** N=674[a]	**Chemo+Surgery** N=715[a]	**p-value**[b]
Clinical Stage I	348 (56%)	272 (44%)	<0.001
Unknown	4	5	
Clinical Stage			<0.001
Stage I	348 (56%)	272 (44%)	
Stage II	187 (44%)	236 (56%)	
Stage III	135 (40%)	202 (60%)	
Unknown	4	5	
Pathologic Stage			<0.001
Stage I	24 (23%)	82 (77%)	
Stage II	15 (11%)	118 (89%)	
Stage III	14 (4.3%)	310 (96%)	
Stage IV	7 (14%)	42 (86%)	
Unknown	614	163	

[a]n (%) row-wise

[b]Pearson's Chi-squared test

.

Caption: This table demonstrates various tumor stage categorized by receipt of chemotherapy or chemotherapy with surgery. *P* values are listed in the last column.

© 2023 Alnajar A et al. *JAMA Network Open.*

eTable 3: Characteristics of MPM Tumors by Treatment Modality (Column-wise Percentages).

**Characteristic**	**Chemo Only** N = 674[a]	**Chemo+Surgery** N = 715[a]	**p-value**[b]
Clinical Stage, n (%)			<0.001
Stage I	348 (52)	272 (38)	
Stage II	187 (28)	236 (33)	
Stage III	135 (20)	202 (28)	
Unknown	4	5	
Pathologic Stage, n (%)			<0.001
Stage I	24 (40)	82 (15)	
Stage II	15 (25)	118 (21)	
Stage III	14 (23)	310 (56)	
Stage IV	7 (12)	42 (7.6)	
Unknown	614	163	
Histological Subtype, n (%)			0.30
Biphasic mesothelioma	113 (17)	135 (19)	
Epithelioid mesothelioma	561 (83)	580 (81)	

[a]n (%) column-wise

[b]Pearson's Chi-squared test

Caption: This table demonstrates various tumor characteristics including histologic subtype, clinical stage, and pathologic stage categorized by receipt of chemotherapy or chemotherapy with surgery. *P* values are listed in the last column.

© 2023 Alnajar A et al. *JAMA Network Open.*

eTable 4: Treatment Specifications and Adjunct Treatments Stratified by the Treatment Modality.

**Characteristic**	**Chemo Only** N = 674[a]	**Chemo+Surgery** N = 715[a]	**p-value**[b]
Treatment Modality			NA
Chemo Only	638 (95)	0 (0)	
Chemo and Radiation	28 (4.2)	0 (0)	
Chemo and Surgery	0 (0)	527 (74)	
Trimodality	0 (0)	170 (24)	
Other	8 (1.2)	18 (2.5)	
Adjunct Treatments, n (%)			0.071
Experimental	12 (1.8)	22 (3.1)	
Immunotherapy	20 (3.0)	14 (2.0)	
Other	641 (95)	673 (94)	
Chemotherapy initiation (days from diagnosis), Median (IQR)	40 (27 – 64)	57 (36 – 92)	<0.001
Unknown	35	56	
Surgery Technique, n (%)			NA
Open/Unspecified	0 (0)	509 (87)	
Robotic	0 (0)	3 (0.5)	
VATS	0 (0)	70 (12)	
Unknown	0 (0)	133	

[a]n (%) column-wise; Median (IQR)

[b]Pearson's Chi-squared test; Fisher's exact test; Wilcoxon rank sum test

© 2023 Alnajar A et al. *JAMA Network Open.*

eTable 5: Univariable Cox (Unadjusted) Model for Factors Correlated with Overall Survival.

**Characteristic**	**HR (95% CI)[a]**	**p-value**
Age		
18-45	1 [Reference]	
45-54	1.43 (0.88-2.32)	0.15
55-64	1.71 (1.10-2.67)	0.017
65+	2.41 (1.56-3.73)	<0.001
Sex		
Female	1 [Reference]	
Male	1.65 (1.43-1.90)	<0.001
Race and/or Ethnicity		
Non-Hispanic White	1 [Reference]	
Hispanic	0.83 (0.63-1.10)	0.19
Asian	1.35 (0.74-2.44)	0.32
Black	1.80 (1.31-2.46)	<0.001
FPL≤150%	1.19 (1.06-1.34)	0.003
Rural areas	1.16 (0.77-1.73)	0.48
Hospital Distance (mi)		
0 to 12.49	1 [Reference]	
12.5 to 249	0.97 (0.86-1.10)	0.67
>250	0.54 (0.39-0.74)	<0.001
Low Educational Attainment	1.21 (1.06-1.37)	0.004
Insurance Status		

© 2023 Alnajar A et al. *JAMA Network Open.*

**Characteristic**	**HR (95% CI)[a]**	**p-value**
Private Insurance	1 [Reference]	
Medicaid/Not Insured	1.21 (0.87-1.69)	0.25
Medicare/Other Government	1.44 (1.27-1.63)	<0.001
Year of Diagnosis		
2004-2008	1 [Reference]	
2009-2013	0.78 (0.68-0.91)	0.001
2014-2017	0.70 (0.59-0.83)	<0.001
Low Hospital Volume	1.25 (1.11-1.41)	<0.001
Facility Type		
Academic	1 [Reference]	
Non-academic Facilities	1.53 (1.34-1.73)	<0.001
Integrated	1.21 (0.99-1.50)	0.067
Clinical Stage		
Stage I	1 [Reference]	
Stage II	1.00 (0.88-1.15)	0.95
Stage III	0.94 (0.81-1.09)	0.41
Therapy Modality		
Chemo Only	1 [Reference]	
Chemo+Surgery	0.57 (0.51-0.64)	<0.001
CDCI Score > 0	1.11 (0.97-1.26)	0.12
Chemotherapy initiation (Z score)[b]	0.89 (0.83-0.95)	<0.001

[a]HR = Hazard Ratio, CI = Confidence Interval

© 2023 Alnajar A et al. *JAMA Network Open.*

eFigure 1. Kaplan-Meier Survival Curves Comparing Patients Based on Treatment Options and Surgical Types: A) between patients underwent chemotherapy with (red) and without (blue) curative surgery; B) between underwent EPP (dark blue) and P/D (dark red) as their curative surgery treatment.

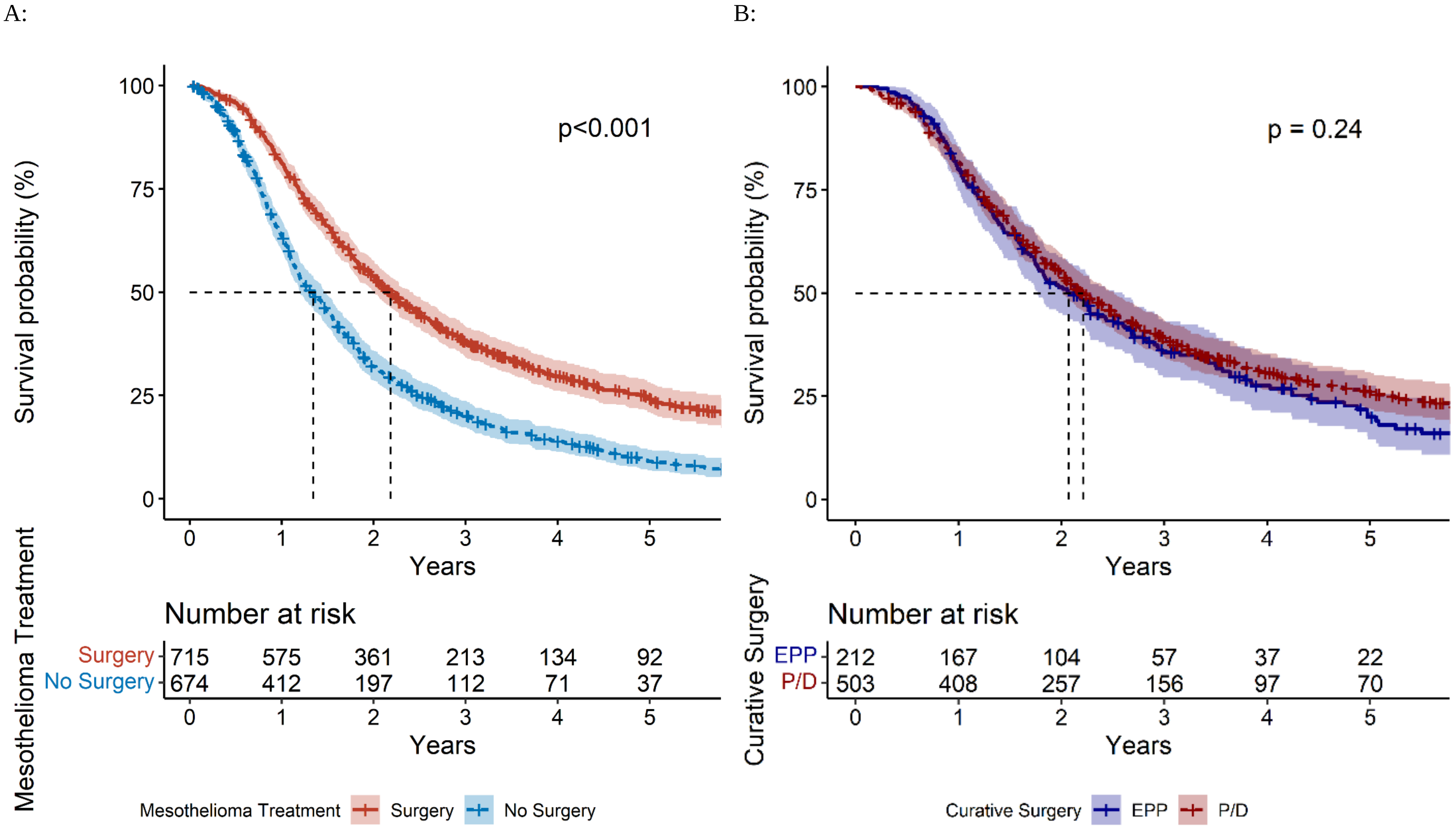

© 2023 Alnajar A et al. *JAMA Network Open.*

eFigure 2: Kaplan-Meier survival curves comparing patients based on race and ethnicity.

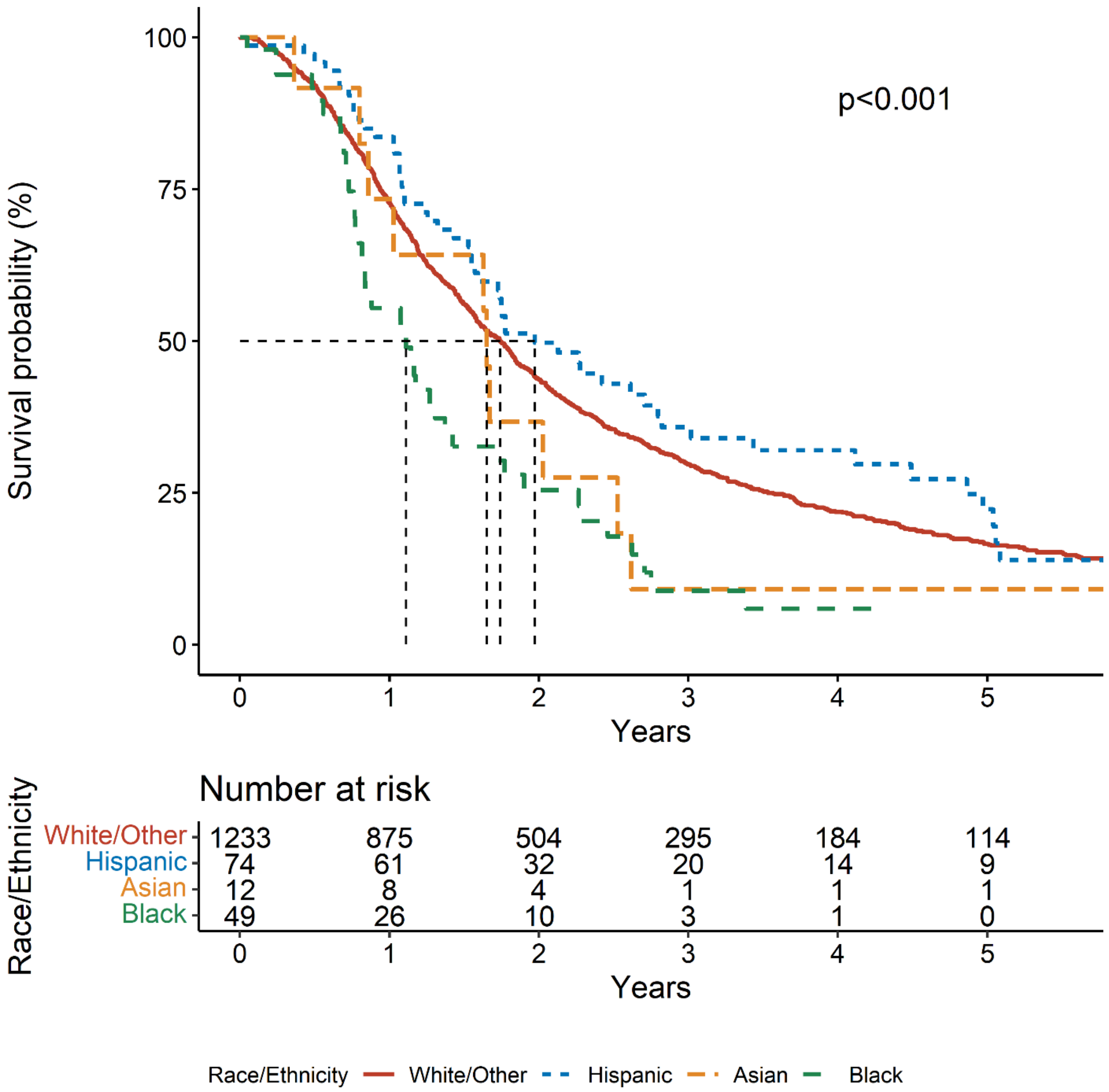

© 2023 Alnajar A et al. *JAMA Network Open.*
