# Supplementary material for: Disparities in Survival Due to Social Determinants of Health and Access to Treatment in US Patients With Operable Malignant Pleural Mesothelioma
Source: JAMA Netw Open. 2023 Mar 23;6(3):e234261. doi: 10.1001/jamanetworkopen.2023.4261 (PMC10037156; doi:10.1001/jamanetworkopen.2023.4261)
Supplement: Supplement 2. — Data Sharing Statement [file jamanetwopen-e234261-s002.pdf]

Data Sharing Statement

Alnajar. Disparities in Survival Due to Social Determinants of Health and Access to Treatment in US Patients With Operable Malignant Pleural Mesothelioma. *JAMA Netw Open*. Published March 23, 2023. doi:10.1001/jamanetworkopen.2023.4261

Data
Data available: No

Additional Information
Explanation for why data not available: NCDB data use agreement don't allow data sharing
